# Reduced Regional Homogeneity in Bilateral Frontostriatal System Relates to Higher Impulsivity Behavior in Codeine-Containing Cough Syrups Dependent Individuals

**DOI:** 10.1371/journal.pone.0078738

**Published:** 2013-11-04

**Authors:** Yingwei Qiu, Xiaofei Lv, Huanhuan Su, Guihua Jiang, Junzhang Tian, Fuzhen Zhuo, Lujun Han, Xuelin Zhang

**Affiliations:** 1 Department of Medical Imaging, Guangdong No. 2 Provincial People's Hospital, Guangzhou, P.R. China; 2 Departments of Medical Imaging and Interventional Radiology, Cancer Center, Sun Yat-Sen University, Guangzhou, P.R. China; 3 Medical Imaging Centre, NanFang Hospital, Southern Medial University, Guangzhou, P.R. China; 4 Addiction Medicine Division, Guangdong No. 2 Provincial People's Hospital, Guangzhou, P.R. China; Institute of Psychology, Chinese Academy of Sciences, China

## Abstract

**Background:**

In the past twenty years, codeine-containing cough syrups (CCS) was recognized as a new type of addictive drugs. However, the exact neurobiologic mechanisms underlying CCS-dependence are still ill-defined. The aims of this study are to identify CCS-related modulations of neural activity during the resting-state in CCS-dependent individuals and to investigate whether these changes of neural activity can be related to duration of CCS use, the first age of CCS use and impulse control deficits in CCS-dependent individuals. We also want to observe the impact of gray matter deficits on these functional results.

**Methodology/Principal Findings:**

Thirty CCS-dependent individuals and 30 control subjects participated. Resting-state functional MRI was performed by using gradient-echo echo-planar imaging sequence. Regional homogeneity (ReHo) was calculated by using REST software. Voxel-based analysis of the ReHo maps between controls and CCS-dependent groups was performed using two-sample t tests (*p*<0.05, corrected for multiple comparisons). The Barratt Impulsiveness Scale 11 (BIS.11) was surveyed to assess participants' impulsivity trait soon after MR examination. Abnormal clusters revealed by group comparison were extracted and correlated with impulsivity, duration of CCS use, and age of first CCS use. ReHo was diminished in the bilateral medial orbitofrontal cortex (mOFC) and left dorsal striatum in CCS-dependent individuals. There were negative correlations between mean ReHo in the bilateral medial OFC, left dorsal striatum and duration of CCS use, BIS.11 total scores, and the subscale of attentional impulsivity in CCS-dependent individuals. There was a significantly positive correlation between mean ReHo in the left dorsal striatum and age of first CCS use in CCS-dependent individuals. Importantly, these results still remain significant after statistically controlling for the regional gray matter deficits.

**Conclusion:**

Resting-state abnormalities in CCS-dependent individuals revealed in the present study may further improve our understanding about the neural substrates of impulse control dysfunction in CCS-dependent individuals.

## Introduction

In the past twenty years, codeine-containing cough syrups (CCS) was recognized as a new type of addictive drugs. Long-term and sustained taking CCS would lead to physical and psychological dependence [1]. As an over-the-counter drug, CCS has become one of the most popular drugs of abuse in young people worldwide for its easy access, and abuse of CCS is increasingly perceived as a problem by modern society [2].

Previous studies had revealed the behavior, personality and mental were abnormal in CCS-dependent individuals [3], [4], which were similar to other illicit substance dependent. By investigating 224 CCS-dependent individuals, Wang et al. showed the CCS had a mainly negative impact on their mental health, with impulsivity, compulsion, depression and anxiety sharing the most prominent factors [5]. However, the exact neurobiological mechanisms underlying these psychological alterations are still ill-defined. To our best knowledge, only one neuroimaging study existed pertaining to CCS-dependent, by employing single photon emission computed tomography (SPECT), Hou et al. demonstrated the dopamine transporter (DAT) availability of striatum as well as volume, weight, and 99mTc-TRODAT-1 uptake ratio of corpus striatum/the whole brain were significantly reduced in CCS-dependent individuals compared with controls [6].

Functional magnetic resonance imaging (fMRI) provided a primary method of mechanism detection of illicit substance abuse in the past two decades. And large body of fMRI studies, administered during the performance of diverse tasks, had suggested the disorganization of the reward, impulse control and memory circuits in illicit substance abuse [7]–[9]. As a new branch of fMRI, “Resting state” fMRI (rs-fMRI) which can assess resting-state brain physiology in the context of clinical studies while avoiding performance confounds attracted more attention in the past ten years [10]. Rs-fMRI was easier to implement than positron emission tomography (PET)/single photon emission computed tomography (SPECT) for its lower cost, greater availability, and noninvasiveness [11]. Low-frequency fluctuations (LFF; <0.08 Hz) of rs-fMRI signals were considered to be related to spontaneous neuronal activity in nonhumans [12], [13] and humans [14], [15]. The amplitude or the synchronous of LFF would change in psychiatric state [16]. Accumulate rs-fMRI studies have suggested that patients with illicit substance abuse, such as heroin [17], [18] and cocaine [19], [20], exhibit decreased LFF synchronous and/or amplitude in particular brain regions.

More recently, the regional homogeneity (ReHo) method [21] was developed to analyze the similarities of intra-regional spontaneous low-frequency (<0.08 Hz) blood oxygenation level-dependent (BOLD) signal fluctuations in voxel-wise analysis across the whole brain. ReHo analysis assumed that, within a functional cluster, the hemodynamic characteristics of every voxel would be similar or synchronous with those of each other, and such similarity could be changed or modulated by different conditions. Kendall's coefficient of concordance (KCC) was used to measure the similarity of the time series of one voxel with those of its nearest neighbors in a voxel-wise analysis. ReHo reflects the temporal synchrony of the regional BOLD signal, which may be potentially helpful to understand the pathophysiology of substance abuse. This method has been used to explore regional neural activity patterns in healthy volunteers [22] and illicit substance dependent individuals [18] in the resting-state.

Impulsivity is a personality trait that presents in healthy individuals, yet the substance-dependent individuals would show higher impulsivity [23], [24]. Previous study had shown that the impulsivity trait was related to the severity of cocaine abuse and severity of cocaine withdrawal symptom in cocaine dependent individuals. Furthermore, cocaine dependent individuals with high impulsivity were likely to drop out of treatment [23], [24]. Higher impulsivity trait was also observed in CCS-dependent individuals in previous clinical and psychological research [4], [5]. Thus, Barratt Impulsiveness Scale 11 (BIS.11) was surveyed to assess participants' impulsivity trait soon after MR examination.

The first purposes of present study are to identify CCS-related modulations of neural activity in the resting-state by using rs-fMRI and ReHo method, and to investigate their correlations with the duration of CCS use, age of first CCS use and impulsivity traits in CCS-dependent individuals.

In addition to the functional deficits, previous studies also indicated illicit substances dependent individuals had lower gray matter volume/density than matched controls [25], [26]; the CCS dependent may have the similar effect. The lower gray matter volume/density may lead to artificial reduction in measured functional signals. While comparing functional differences between CCS-dependent individuals and normal controls, this issue could potentially be crucial due to individual and group differences in the regional gray matter volume. Hence, determining the impact of regional gray matter deficits on the functional results is the other purpose of this study.

## Materials and Methods

### Participants

This prospective study was approved by the Research Ethics Review Board of the Institute of Mental Health at the Guangdong No. 2 Provincial People's Hospital. Written informed consent was obtained from all subjects. Sixty subjects, including 30 (50%) control subjects and 30 (50%) CCS-dependent individuals participated in this study. The CCS-dependent individuals were randomly selected from the patients seeking treatment at Addiction Medicine Division of Guangdong No. 2 Provincial People's Hospital. Patients were screened based on the DSM-IV criteria from the medical history, along with a urine test and an interview. They regularly used cigarettes and denied any use of psychotropic agents in the month prior to the rs-fMRI scan being performed. Inclusion criteria for the control subjects were lack of diagnosis of substance abuse or dependence. Exclusion criteria for all participants included neurological illness, schizophrenia or bipolar disorder, prior significant head trauma, positive HIV status, diabetes, Hepatitis C, other major medical illness and left-handedness.

### Impulsivity assessment

Impulsivity was assessed using the BIS.11, which is one of the oldest and most widely used self-report measures of impulsive personality traits. This 30-item self-rated scale has three oblique factors: attentional/cognitive, which measures toleration for cognitive complexity and persistence; motor, which measures the tendency to act on the spur of the moment; and nonplanning impulsivity, which measures the lack of sense of the future. Items are rated from 1 (rarely/never) to 4 (almost always/always). To determine overall impulsiveness scores, all items were summed, with higher scores indicating greater impulsivity [27]. BIS.11 is a valid and reliable instrument for Chinese healthy and psychiatric populations [28].

### MRI scanning

MRI data were obtained on a Philips Achieva 1.5T Nova dual MR scanner using a 16 channel NV coil in the Department of Medical Imaging, Guangdong No. 2 Provincial People's Hospital. None of the subjects were taking any medications at the time of the scans. Tight but comfortable foam padding was used to minimize head motion, and ear plugs were used to reduce scanner noise. Rs-fMRI scans were performed by an echo planar imaging (EPI) sequence with scan parameters of repetition time (TR)  = 2000 ms, echo time (TE)  = 50 ms, flip angle  = 90°, matrix = 64×64, field of view (FOV)  = 230×230 mm^2^, slice thickness = 4.5 mm and slice gap = 0 mm. Each brain volume comprised 22 axial slices and each functional run contained 240 volumes (8 minutes) (the slices were approximately along the AC-PC line and covered about −30 to 60 in the IS direction), during resting state fMRI scanning, subjects were instructed to close their eyes and keep still as much as possible, and not to think of anything systematically or fall asleep. Sagital structural images (160 sagital slices, TR  = 25 ms, TE  = 4.1 ms, thickness  = 1.0 mm, no gap, in-plane resolution  = 231×232, FOV = 230×230 mm, flip angle  = 30°) were acquired using a fast field echo (FFE) three-dimensional T1 weighted sequence.

After MR examination, all the participants were asked some questions to verify the degree of their cooperation.

### Data processing and ReHo calculation

The imaging data were mainly preprocessed with SPM8 (http://www.fil.ion.ucl.ac.uk/spm). For each participant, the first ten time points were discarded to avoid transient signal changes before magnetization reached steady-state and to allow subjects to get used to the fMRI scanning noise. The raw data were corrected for temporal shifts between slices, corrected for head motion (a least squares approach and a 6 parameter spatial transformation). Subjects with head motion exceeding 1.0 mm of maximal translation (in any direction of x, y or z) or 1.0° of maximal rotation through the resting-state run were discarded from further analysis. Moreover, we also performed a two-sample t test on the mean absolute estimated movement parameters (shift and rotation) on all three axes to examine between-group differences in the degree of head motion. Following the motion correction, all data were spatial normalized to the Montreal Neurological Institute (MNI) template (resampling voxel size  = 3×3×3 mm^3^). Then all images spatially normalized to the MNI template were transformed to Talairach and Tournoux coordinates.

Further data preprocessing and ReHo analysis were performed with REST software (http://resting-fmri.sourceforge.net) [29]. Subsequent data preprocessing included removal of linear trends and temporally filtered (band pass, 0.01–0.08 Hz) to remove the effects of very low-frequency drift and high-frequency noises (e.g., respiratory and cardiac rhythms) [30], [31]. The ReHo calculation procedure: The calculation procedure was the same as that reported in the previous studies [21]. In brief, this was accomplished on a voxel-by-voxel basis by calculating KCC [32] of time series of a given voxel with those of its nearest 26 neighbors (see formula below). A larger value for a given voxel indicated a higher regional homogeneity within a cluster made up of the voxel and its nearest neighbors.



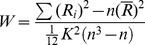
(1)where W is the KCC among given voxels, ranging from 0 to 1; R_i_ is the sum rank of the ith time point (R_i_ = 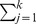
r*_ij_* where r*_ij_* is the rank of the *i*th time point in the *j*th voxel); 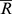 = ((n+1) K)/2 is the mean of the R_i_; *k* is the number of time series within a measured cluster (27, one given voxel plus the number of its neighbors); and *n* is the number of ranks (here, n = 230). The KCC value was calculated to this voxel, and an individual KCC map was obtained for each subject. To reduce the influence of individual variations in the KCC value, normalization of regional homogeneity maps was preformed through dividing the KCC among each voxel by averaged KCC of the whole brain. The resulting data were then spatially smoothed with an 8-mm full-width at half-maximum (FWHM) Gaussian kernel to reduce noise and residual differences in gyral anatomy.

### Measurements of regional brain gray matter deficits

In order to investigate the impact of regional brain gray matter deficits on the functional results, we calculated a regional gray matter index of each region showing significant between-group ReHo difference as follows. First, the Talairach-normalized and re-sampled (1 mm^3^) 3D anatomical images of each subject within the brain mask were automatically segmented into gray matter, white matter and cerebrospinal fluid (CSF) by using a DARTEL method [33]. In the next step the regions exhibiting significant between-group ReHo differences were defined as group regions of interest (ROIs). The regional gray matter index of each region was calculated by dividing the gray matter volume of the corresponding ROI by the total volume of individuals' brain gray matter.

### Statistics

Two-sample t tests were performed to assess the differences in age, duration of education, cigarette smoking, head motion and regional gray matter index between CCS-dependent individuals and control groups, Pearson Chi-Square test was performed to assess the differences in gender between the two groups using SPSS (version 13.0), and a *p*<0.05 (two-sided) was deemed significant.

To explore the ReHo differences between the CCS-dependent individuals and controls, a second-level random-effect two-sample t-test was performed on the individual normalized ReHo maps in a voxel-by-voxel manner with head motion as covariate [18], [34], *p*<0.05 (corrected for multiple comparisons) was considered to show significant differences between the two groups. Threshold correction was done by using the AlphaSim program in AFNI, which applies Monte Carlo simulation to calculate the probability of false positive detection by taking into consideration both the individual voxel probability thresholding and cluster size [35]. Using this program, a corrected significance level of *p*<0.05 was obtained by clusters with a minimum volume of 2106 mm^3^ at an uncorrected individual voxel height threshold of *p*<0.01. (Parameters were: individual voxel *p* value = 0.01, 1,000 simulations, FWHM = 8 mm, with brain mask). Moreover, to identify the association between the alteration of ReHo, the duration of cough syrup usage, and age of first cough syrups use. The average ReHo values of all voxels in the reduced areas revealed by voxel-based analysis were extracted separately using the extract time series in REST and were input into SPSS. Then, bivariate correlation was introduced to indicate the relationships between the ReHo values of the significant differences in the CCS-dependent individuals with the duration of cough syrup usage, age of first cough syrups use, and BIS.11 scores, the significance levels were set at *p*<0.05 (two-tailed, corrected for multiple comparison).

To determine whether the functional results were influenced by the structural deficits, we further examined the between-group differences in mean ReHo using a two-sample t test by taking individual regional gray matter indices as covariates. Additionally, the correlation of the mean ReHo and the duration of cough syrup usage, age of first cough syrups use, and BIS.11 scores in the CCS-dependent individuals were also examined by a bivariate analysis with individual regional gray matter indices as covariate. Finally, the statistical significances were compared with the functional results obtained from the same processes but without accounting for the regional gray matter deficits.

## Results

**Figure 1:**
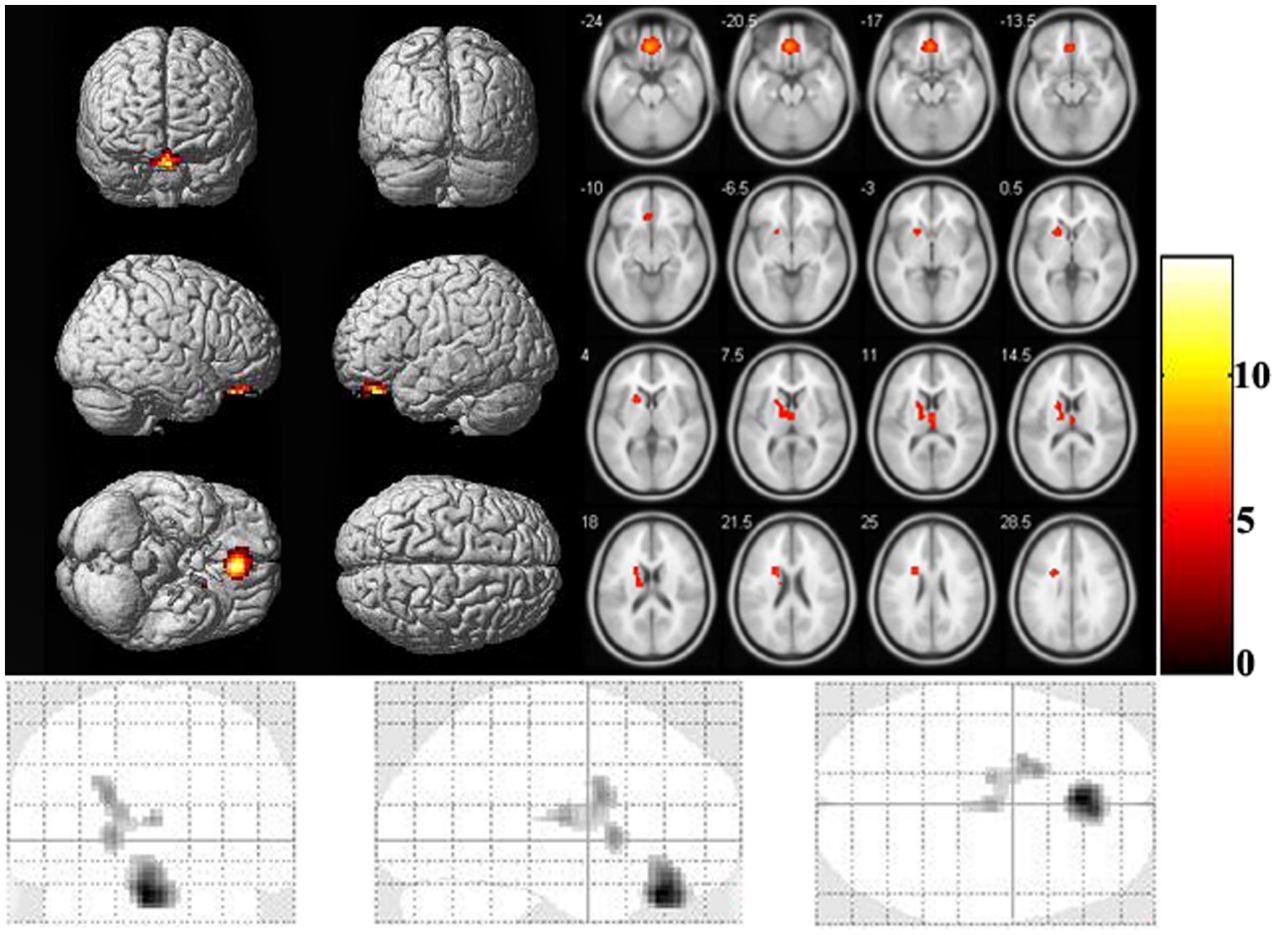
Brain areas with abnormal regional homogeneity in CCS-dependent individuals when compared with control groups. The differences show in whole-brain MR rendering (top left), MR axial view (top right), and coronal (bottom left), sagittal (bottom middle), and axial (bottom right) glass brain map at the given threshold (cluster size >2106 mm^3^, T>2.7479 (or T<−2.7479) and P<0.01, correlated for p<0.05).

There were no significant differences in age, gender, education, number of cigarette smoked per day, and head motion between the CCS-dependent individuals and controls (Table 1). Compared with the normal controls, the CCS-dependent group showed significant reduced ReHo in the bilateral medial OFC (extent to bilateral anterior cingulate cortex) and left dorsal striatum (extent to bilateral thalamus) (two-sample t test, *p*<0.05, corrected; Fig. 1 and Table 2). There were no areas with increased ReHo in CCS-dependent individuals. As gender can affect the brain ReHo index in healthy subjects [36], which may also affect the present study, thus we also compared the 28 male CCS-dependent individuals with the 28 male controls. However, a similar result was produced.

**Table 1:** Demographic and Clinical Characteristics of the codeine-containing cough syrups dependent (CCS-dependent) individuals and Control subjects.

*Characteristic*	*CCS-dependent individuals (n = 30)*	*Control subjects (n = 30)*	*Z Value*	*p value*
Age (years)	25.07 (3.08)^a^	23.97(2.47) a	2.706	0.133
Gender (male:female)	28:2	28:2	-	1.000
Education (years)	13.03 (2.76) a	12.07 (3.42) a	1.126	0.233
Nicotine ((no. cigarette/day))	16.53 (9.61) a	13.50(8.82) a	0.036	0.208
Cough syrups use (years)	5.08 (range:1∼8)	N/A^b^	-	-
Age of first use cough syrups	19.93 (range:12∼30)	N/A^b^	-	-
Mean dose (ml/d)	487.33(range:60∼1800)	N/A^b^	-	-
Head motion				
Shift	12.32 E-2(1.64 E-2)	12.19 E-2(1.42 E-2)	1.372	0.354
Rotation	1.69 E-3(0.52 E-3)	1.71 E-3(0.66 E-3)	−0.772	0.425
Total BIS scores	71.63(4.60) a	57.13(5.18) a	0.778	0.000^ 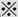 ^
Attentional impulsivity	18.27(2.95) a	15.57(1.63) a	5.341	0.000^ 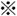 ^
Motor impulsivity	30.50(2.74) a	22.13(3.38) a	2.727	0.000^ 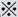 ^
Non-plan impulsivity	22.87(1.96) a	19.63(2.71) a	1.755	0.000^ 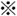 ^

a : means±standard deviations. ^b^: N/A = not applicable. ^
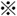
^: *p*<0.05.

**Table 2:** Brain Regions with Abnormal (decrease) Regional Homogeneity in CCS-dependent individuals compared with Controls Subjects.

*Brain areas*	*Side*	*MNI* a *coordinate*			*BA^c^*	*Peak t values*	*Volume (mm^3^)*
		*x*	*y*	*z*			
Medial OFC	R/L^b^	−3	36	−24	11, 32	7.909	7560
Dorsal striatum	L^b^	−15	−9	15	-	4.774	3078

a : MNI, Montreal Neurological Institute; ^b^: L, left; R, right. ^c^: BA, Brodmann area.

Comparison of average BIS.11 scores in the CCS-dependent group and control group was showed in Table 1. Compared to the control group, we observed significantly higher attentional impulsivity, motor impulsivity, non-plan impulsivity and total scores for the CCS-dependent group (*p*<0.05).

Significantly negative correlation was observed between the mean ReHo values in dorsal striatum which showed group differences and the duration of CCS use in CCS-dependent individuals and significantly positive correlation was observed between the mean ReHo values in dorsal striatum which showed group differences and the age of first CCS use in CCS-dependent individuals (*p*<0.05/2, Bonferroni correction; Table 3). Significantly negative correlation was observed between the mean ReHo values of the bilateral medial OFC, bilateral dorsal striatum and the BIS.11 total scores, the subscale of attentional impulsivity in both CCS-dependent individuals and controls (*p*<0.05/2, Bonferroni correction; Table 4).

**Table 3:** Correlation between Mean Regional Homogeneity of frontostriatal system that showed group different and duration of cough syrups use, age of first cough syrups use.

	*MNI* a *coordinate*			*Age of first CCS use*		*Duration of CCS use*	
	x	y	z	cc^b^	*p* value	cc^b^	*p* value
Medial OFC	−3	36	−24	0.378	0.039	−0.38	0.038
Dorsal Striatum	−15	−9	15	0.417	0.022^ 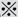 ^	−0.411	0.024^ 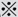 ^

a : MNI, Montreal Neurological Institute. ^b^: **cc**, correlation coefficient. ^
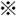
^: *p*<0.05/2 (Bonferroni correction).

**Table 4:** Correlation between Mean Regional Homogeneity of Abnormal Brain Regions and Total BIS scores, Attentional impulsivity subscale, Non-plan impulsivity subscale and Motor impulsivity subscale in control and CCS-dependent individuals.

	*Control*		*CCS-dependent individuals*	
	cc	*p* value	cc	*p* value
***Total BIS***				
Medial OFC	−0.407	0.026	−0.458	0.011 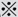
Dorsal striatum	−0.468	0.009 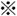	−0.544	0.002 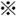
***Attentional***				
Medial OFC	−0.438	0.016 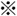	−0.214	0.257
Dorsal striatum	−0.460	0.010 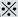	−0.576	0.001 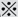
***Non-plan***				
Medial OFC	−0.072	0.707	−0.325	0.080
Dorsal striatum	−0.302	0.105	−0.157	0.407
***Motor***				
Medial OFC	−0.443	0.014 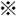	−0.266	0.155
Dorsal striatum	−0.199	0.293	−0.165	0.385

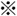
: *p*<0.05/2 (Bonferroni correction).

### Examination of regional gray matter deficits

**Figure 2:**
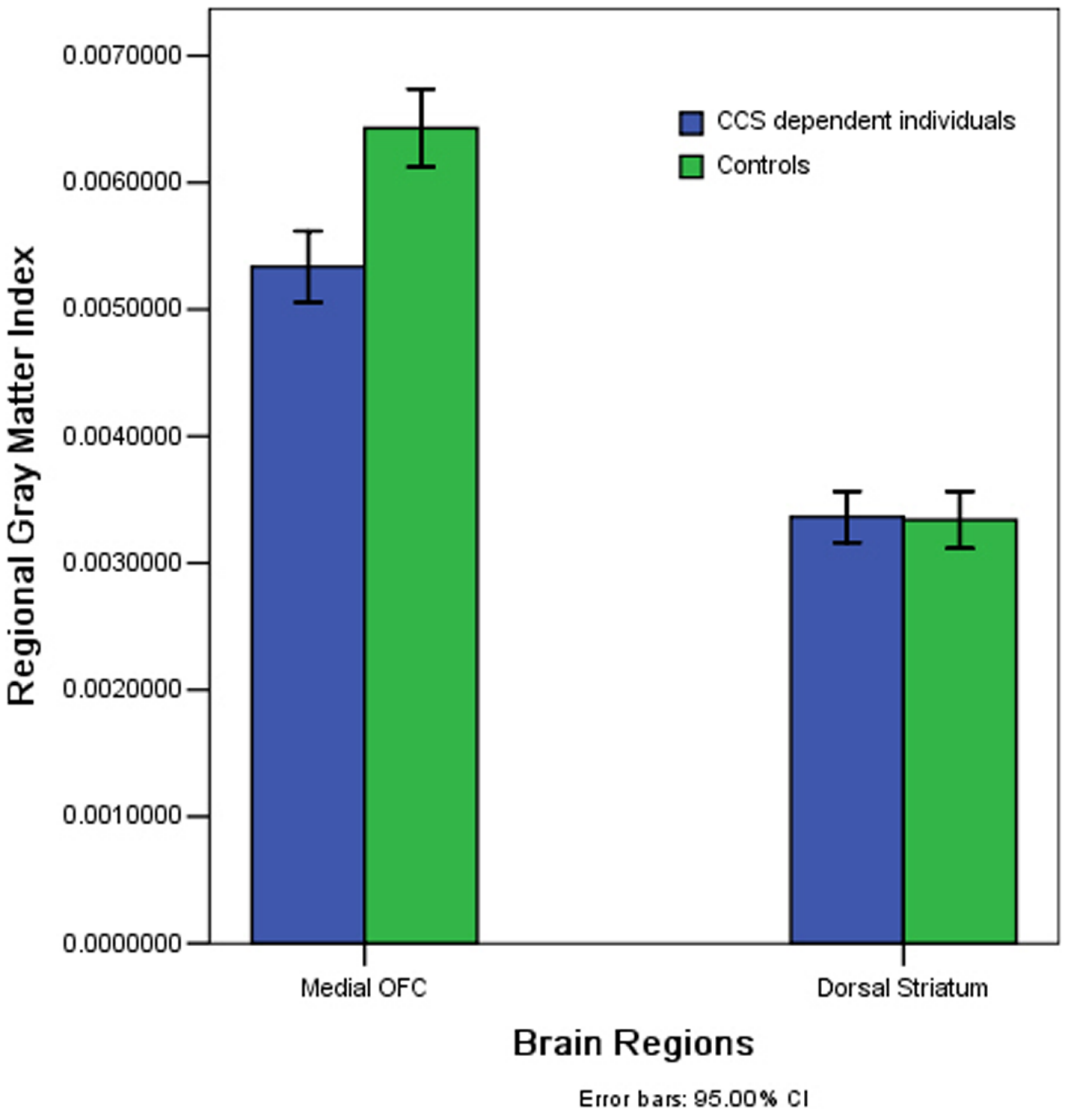
Mean regional gray matter index with standard error bars of each region in CCS-dependent individuals versus normal controls. Significantly lower gray matter volume was observed in the CCS-dependent individuals in the bilateral medial OFC (P<0.001) but not in the left dorsal striatum (P = 0.872).

The CCS-dependent individuals had lower gray matter volume than normal controls in the bilateral OFC (t = −5.368, P<0.001) (Fig. 2). There was no significant difference observed in the left dorsal striatum (t = 0.161, P = 0.872) (Fig. 2).

### Effect of regional gray matter deficits on ReHo analysis

Bilateral medial OFC demonstrated both significant ReHo decreases and gray matter deficits. After controlling for the regional gray matter deficits by statistical analysis (the regional gray matter index was used as covariate), significant bilateral medial OFC ReHo differences (F = 54.353, p = 7.62E-10) between the two groups and significant correlation of bilateral medial OFC ReHo against the BIS.11 total scores (t = −0.432, p = 0.019) in the patients group was still observed. However the statistical significance was reduced compared to the results without accounting for the regional gray matter deficits with (t = −10.247, p = 1.24876E-14) for the ReHo differences, and (t = -0.458, p = 0.011) for the correlation analysis.

### Post-hoc check for susceptibility artifact signal loss

**Figure 3:**
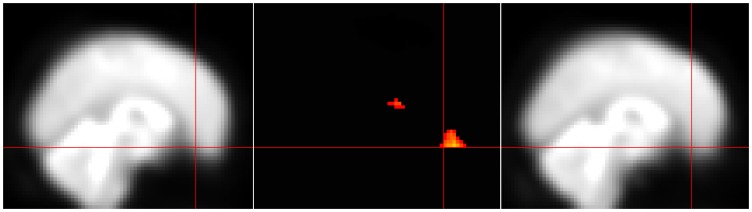
Both groups show adequate T2* signal in the region of the medial OFC. The region of the medial OFC is often prone to loss of T2* signal due to susceptibility artifact. Here it is shown that the scanning parameters used in this study allowed for adequate signal detection in this region that does not appear to differ between the groups. The mean T2* images from the 30 controls (right panel) and the 30 CCS-dependent individuals (right panel) are shown with the crosshairs on the medial OFC cluster (middle panel) that showed lower ReHo in CCS-dependent individuals.

Given the detection of signal differences between groups in the bilateral medial OFC, a region prone to fMRI signal loss [37], we have included a qualitative comparison of the mean T2* signal in this region. To do so, we created mean T2* maps for each group by adding 1 volume (the twentieth) from each subject's time series and dividing by the number of subjects. The mean T2* image for each of the two groups is shown in Figure 3 along with the bilateral medial OFC cluster to demonstrate that there was adequate signal in this region.

## Discussion

To our best knowledge, this is the first study to investigate the ReHo of brain spontaneous activity in CCS-dependent individuals. Compared with the controls, reduced ReHo was found in prefrontal-striatal system, including bilateral medial OFC (extent to bilateral anterior cingulate cortex) and left dorsal striatum (extent to bilateral thalamus). No increased ReHo was found in CCS-dependent individuals. We further found that the mean ReHo value in bilateral mOFC and left dorsal striatum revealed by group comparison was associated with total BIS.scores and the subscale of attentional impulsivity in CCS-dependent individuals. The mean ReHo value in left dorsal striatum was related to the duration of CCS use, age of first cough use in CCS-dependent individuals, i.e. the longer (younger) the CCS-dependent individuals have been using (start to use) CCS, the lower of the mean ReHo in this region. Most importantly, these results still remain significant after statistically controlling for the regional gray matter deficits. Thus, the present investigation expands our understanding of CCS abuse on the brain function in human.

The medial OFC (BA10) is a part of the limbic system which involves ongoing monitoring of the reward value of a reinforcer [38]. In present study, we found local synchronization of spontaneous low-frequency BOLD fluctuation was destructed in bilateral medial OFC, which implied functional deficits of these regions. This finding was agreed with previous neuroimaging studies related to illicit drug abuse. In a SPECT study, Botelho et al. reported a decreased brain perfusion in the frontal cortex, mainly the OFC, as well as in the occipital and temporal regions, basal ganglia, and cerebellum in heroin dependent individuals with 10 weeks of detoxification [39]. By employing positron emission tomography (PET) and a dual-tracer approach, Volkow et al [40] revealed decreased metabolism in several regions of the frontal lobes, most markedly the OFC and cingulate gyrus in cocaine users when compared with healthy control subjects. And in task-state fMRI studies, with diverse cues, the mOFC and DLPFC showed abnormal activation in illicit drug dependent individuals, with accentuated in drug-related cues [9], [41]–[44]and attenuated in non drug-related cues [9], [45]. Recently, by employing rsfMRI, Ma et al. demonstrated the significantly stronger functional connectivity between mOFC and Nucleus accumbens, between mOFC and lateral OFC and within the mOFC as well as weaker functional connectivity between dlPFC and lateral OFC, between dlPFC and dACC in heroin dependent individuals [46]. And our previous studies also showed the amplitude and the local synchronization of the BOLD signal were destructed in bilateral mOFC in heroin dependent individuals [17], [18]. Together with previous neuroimaging studies of illicit substance abuse, our findings demonstrate that the CCS abuse can impair the mOFC just as illicit substance abuse do, which may be the neural mechanism of deficits in salience evaluation in CCS-dependent individuals.

Dorsal striatum forms a continuous and large mass [47], topographically separated by the internal capsule into caudate and putamen. Current evidence suggested that the dorsal striatum contributed directly to decision-making, especially to action selection and initiation [48]. The reduced ReHo in dorsal striatum observed in present study, implying dysfunctional of this region, was consistent to the previous studies which showed decision-making deficits in CCS-dependent individuals [4], [22]. And the dysfunctional of dorsal striatum uncovered in present study also agreed with a recent study of CCS abuse. By employing SPECT, Hou et al. demonstrated that DAT availability of striatum as well as volume, weight, and 99mTc-TRODAT-1 uptake ratio of corpus striatum/the whole brain were significantly reduced in CCS-dependent subjects when compared with controls [6]. Atypical function in the dorsal striatum was also implicated in previous studies of patients with chronic illicit substance abuse. PET studies showed that addicted subjects had significant reductions in dopamine receptors D_2_ (D2R) availability in striatum that persisted months after protracted detoxification [49]. Structural MRI studies had revealed the gray matter volume of dorsal striatum was lower in illicit substance dependent individuals when compared with controls [50]. And recently, different fMRI studies also showed the dysfunctional of striatum was related to motivational, cognitive and motor deficits in illicit substance dependent individuals [51], [52].

Taken together, we demonstrate that CCS abuse can destroy the brain function of the addicts as other illicit drugs do, especially for prefrontal-striatal system, including the bilateral mOFC and left dorsal striatum. This may raise community awareness of CCS abuse, and help rectify the false beliefs that the cough syrups are non-addictive and less harmful [53]. In view of the relatively lack of preventive education and publicity materials on anti-cough syrups abuse, this result may promote government to review the existing anti-cough medicine initiatives regarding preventive education and prevention.

### Correlation between the ReHo index and BIS scores

Consistent with previous studies about illicit drug addiction, the CCS-dependent individuals scored significantly higher on the impulsivity questionnaires when compared with healthy volunteers. It is known that high scores on BIS.11 imply on increased tendency to shift attention quickly causing inappropriately rapid decision making, acting without thinking and failure to consider the future implication of behavior [27]. The higher BIS scores observed in CCS-dependent individuals in present study agreed with the higher impulsivity observed in CCS-dependent individuals.

Interestingly, the mean ReHo value in bilateral mOFC showing group differences was negatively correlated with BIS scores in both CCS-dependent individuals and controls, which was consistent to previous studies declaring the mOFC was functional for impulse control. In a structural MRI study, Matsuo et al. demonstrated the lower OFC volume was associated with the higher impulsivity in healthy people [54]. Thus the lower ReHo in bilateral OFC, which implies dysfunctional of the OFC, may be the neural mechanisms of the higher impulsivity in CCS-dependent individuals. Though that the causation between the lower ReHo of OFC and the higher impulsivity in CCS-dependent individuals is still needed to be asserted in future study. This hypothesis was also confirmed by previous study of illicit-substance dependent. In a structural MRI study, Ersche et al. demonstrated the GM volume was lower in OFC in cocaine, which was negatively associated with aspects of impulsivity and compulsivity in patients [50].

We also observed a negative correlation between the mean ReHo in left dorsal striatum (extent to bilateral thalamus) and the subscale of attentional impulsivity in CCS-dependent individuals, which means the lower mean ReHo in left striatal, the more rapid shifts in attention and more impatient with complexity in patients. This finding declared the higher attentional impulsivity directly related with dorsal striatum dysfunction. This phenomenon was also observed in attention deficit hyperactivity disorder (ADHD) patients, who are characterized primarily by the co-existence of attentional problems and hyperactivity. In an rs-fMRI study, Cao et al. also found boys with ADHD showed decreased ReHo in the frontal- striatal-cerebellar circuits [55]. Thus the decreased ReHo in left dorsal striatum may be the neural mechanisms of the higher attentional impulsivity in CCS-dependent individuals

### Correlation between the ReHo index and clinical variables

A negative correlation was observed between duration of cough syrup use and mean ReHo value in the left dorsal striatum in CCS-dependent individuals. In other words, greater duration of CCS use was associated with relatively greater destruction of local synchronization of spontaneous low-frequency blood oxygenation in this structure. Previous neuroimaging studies related to illicit drug addiction also demonstrated this effect. For example, Yuan et al found that the gray matter density of the prefrontal, temporal and insular was negatively correlated with the duration of heroin use in heroin dependent individuals [25]. Ersche et al. demonstrated a strong negative correlation between duration of cocaine use and grey matter volume in frontal and cingulated cortex, insula and caudate in cocaine dependent patients [50]. Our previous study using resting-state fMRI also showed a significantly negative correlation between the duration of heroin usage and mean ReHo in most of regions that showed group different in heroin dependent individuals [18]. Together with previous studies, we declare that CCS have a accumulative effect on brain function as other illicit drug do, hence, early intervention is particularly important for the treatment of CCS addiction.

We also found a modest, but significantly positive correlation between the age of first CCS use and the mean ReHo value in the left dorsal striatum that showed group differences. This meant that the earlier use of CCS, the greater the impact on brain function. This phenomenon was also proved in previous studies in illicit drug addiction, Wison et al revealed subjects who started using marijuana before age 17 had smaller whole brain and percent cortical gray matter than those who started later, and male who started using marijuana before age 17 had significantly higher CBF than others [56]. Given ongoing neuromaturation during youth, adolescents may be more vulnerable to potential consequences of addictive substance use than adults. Taken together, our result suggests that adolescents are more vulnerable to neurofunctional abnormalities associated with chronic addictive use than adults; however, the impact of preexisting risk factors is still unknown.

### Effect of regional gray matter deficits on ReHo analysis

It is important to note here that we took into account the impact of regional gray matter deficits on the functional results (ReHo) shown in the bilateral medial OFC. Numerous studies have demonstrated that addicts have lower gray matter volume/density in the bilateral medial OFC than normal controls [24],[25]. Lower gray matter volume/density may cause a partial volume effect in functional imaging techniques, which has been recognized in previous PET studies [57], [58], and recently, fMRI studies have also attempted to clarify the relationship between regional gray matter deficits and BOLD signals during the performance of tasks [59], [60], also in resting state [61]. In this study, we found that CCS-dependent individuals showed significantly lower gray matter volume in the bilateral medial OFC when compared with normal controls (Fig.2), suggesting the necessity for controlling the regional brain gray matter deficits in the functional analysis. By taking into account the regional gray matter deficits as a covariate, we found that statistical significance was reduced in both between-group differences in the medial OFC and the correlation between the medial OFC ReHo and the BIS.11 total scores. It implies that the CCS-related functional results in the medial OFC can be at least partly explained by regional gray matter deficits.

### Limitations

Some limitations in our study need to be addressed. Firstly, because the present study is a cross-sectional one, the cause-effect relationships among the variables cannot be rigorously assessed. Secondly, the results of BIS.11, as a self-report scale, may not fully reflect the impulsivity of all the subjects for someone may not answer the questions honestly. Thirdly, the CCS-dependent individuals changed dose of CCS abuse over time, so we cannot assess the relationship between the total amounts and the clinical data directly. In the end, methodological issues concerning the use of ReHo should be considered when interpreting these results.

### Conclusion

In conclusion, compared with controls, the CCS-dependent individuals exhibit decreased ReHo in the bilateral frontostriatal brain system progressively, which relate to the first age of CCS use and higher impulsivity trait in CCS-dependent individuals. Importantly, these results still remain significant after statistically controlling for the regional gray matter deficits. Thus this resting-state fMRI study suggests that the decreased ReHo of these regions may implicate the underlying pathophysiology in CCS-dependent individuals.
